# Status Epilepticus in Creutzfeldt-Jakob Disease: A Case Report of an Unusual Presentation

**DOI:** 10.7759/cureus.26470

**Published:** 2022-06-30

**Authors:** Parisha Bhatia, Mona Sonbol, Deepali Jain, Noemi Rincon-Flores, Alfred Frontera

**Affiliations:** 1 Neurology, University of South Florida Morsani College of Medicine, Tampa, USA; 2 Neurology, James A. Haley Veterans Hospital, Tampa, USA

**Keywords:** rt-pcr, epilepsy, cjd, prion disease, dementia

## Abstract

Although non-prion neurodegenerative illnesses are the main causes of rapidly progressive dementia (RPD), a case of RPD should be evaluated for Creutzfeldt-Jakob disease (CJD), a kind of prion disease. We describe a 71-year-old man who first displayed a lack of coordination before developing focal seizures accompanied by myoclonic jerks as well as left hemibody weakness and incoordination. As part of the additional diagnostic workup, cerebrospinal fluid (CSF) analyses, 72 hours of prolonged electroencephalogram (EEG) monitoring, and additional brain imaging were obtained. Cortical ribboning was seen in the magnetic resonance imaging (MRI) of the brain, protein 14-3-3 test in the CSF was normal, lateralized and generalized periodic discharges were seen on the EEG. After the patient was examined for additional causes, such as autoimmune encephalitis and seizures, the diagnosis of likely CJD was made. Ultimately, an autopsy was performed and confirmed the diagnosis of definitive CJD.

## Introduction

Creutzfeldt-Jakob disease (CJD), being a fatal neurodegenerative disease, should be high on the differential for patients who exhibit symptoms of rapidly progressive dementia (RPD). CJD is caused by the accumulation of abnormally folded protease-resistant form of protein in the brain called prion protein (PrP) [1]. Prion diseases can present in three main ways: spontaneous (85% of cases), genetically predisposed, and acquired [1,2]. The typical initial symptoms are neuropsychiatric such as depression and anxiety irritability, and as they get worse, cerebellar ataxia, myoclonus, mutism, and visual abnormalities show up. The literature describes a variety of uncommon manifestations. Although seizures are not typical as a first sign of the condition, they can happen [3]. Atypical presentations in about 10% of CJD cases provide a diagnostic challenge for medical professionals [3]. Atypical manifestations have been documented in the literature, including expressive aphasia, hallucinations, vertigo, unexplained progressive cognitive impairment, and the incredibly unusual status epilepticus [3,4]. We present an interesting case of CJD with the initial presentation of focal seizures with preserved awareness.

## Case presentation

A 71-year-old gentleman with medical comorbidities of Type II diabetes mellitus and hypertension was brought in by the family for one week of left-hand incoordination and intermittent confusion. He allegedly swerved off the road while driving without cause. He arrived at the emergency room hypertensive, with systolic blood pressure in the high 190s. With the exception of considerable dysmetria, while testing finger to nose with his left hand, his neurological examination was normal. Laboratory workup revealed an elevated A1c and a low vitamin D. The remainder was unremarkable. A computerized tomography (CT) scan of the head and a magnetic resonance imaging (MRI) of the brain showed extensive chronic microvascular ischemic changes without any acute intracranial pathology. The workup also included an electroencephalogram (EEG) that showed evidence of moderate to severe encephalopathy without evidence of epileptiform discharges. At this time, his working diagnosis remained acute encephalopathy as his confusion had resolved. He was discharged with incomplete resolution of his left-hand incoordination.

Three weeks post-discharge, his family reported him to have recurrent brief jerking episodes involving his head and upper extremities. These episodes were occasionally accompanied by a forced turn of the head and gaze to the right while maintaining awareness. These events happened numerous times a day, each lasting a few minutes. The patient continued to lose a large amount of weight and experienced sleep difficulties, visual and auditory hallucinations, and increased perplexity. He noticeably lost strength in his left hemibody, which made him prone to falling. Over the course of a few weeks, he underwent a dramatic decrease in his cognitive and functional abilities.

On physical examination, the patient appeared alert, but his memory, attention, and ability to focus were all compromised. He also appeared disoriented and had minor slurred speech. The motor exam showed mildly diminished (4+/5) strength in the left hemibody and increased tone in both arms with cogwheel rigidity. His reflexes demonstrated bilaterally positive Babinski's signs and a positive Hoffman's sign on the left. The sensory exam showed diminished pinprick sensation in the left hemibody. The patient was dragging his left leg and had an erratic stride.

**Figure 1:**
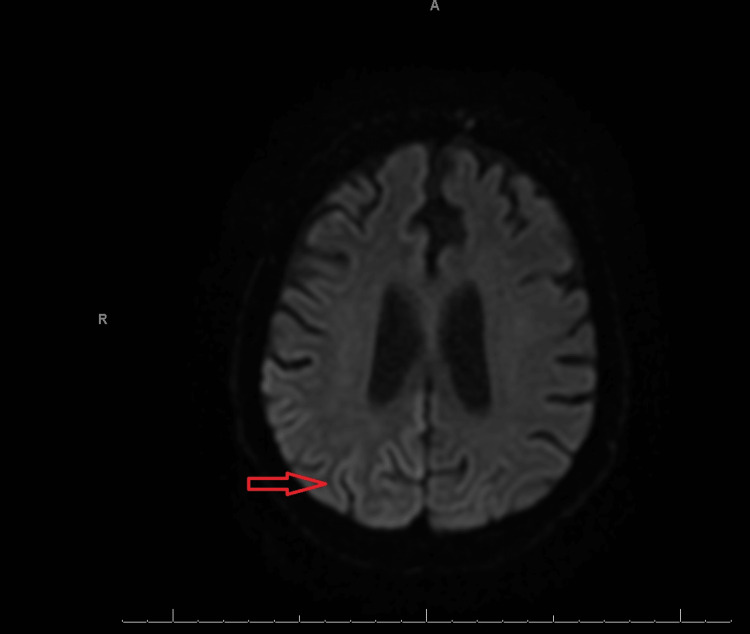
MRI of the brain (DWI) without contrast displaying areas of curvilinear hyperintense signal in the right temporo-occipital cortex (red arrow) Curvilinear hyperintense signal with cortical restricted diffusion is called cortical ribboning. DWI: Diffusion-weighted imaging.

**Figure 2:**
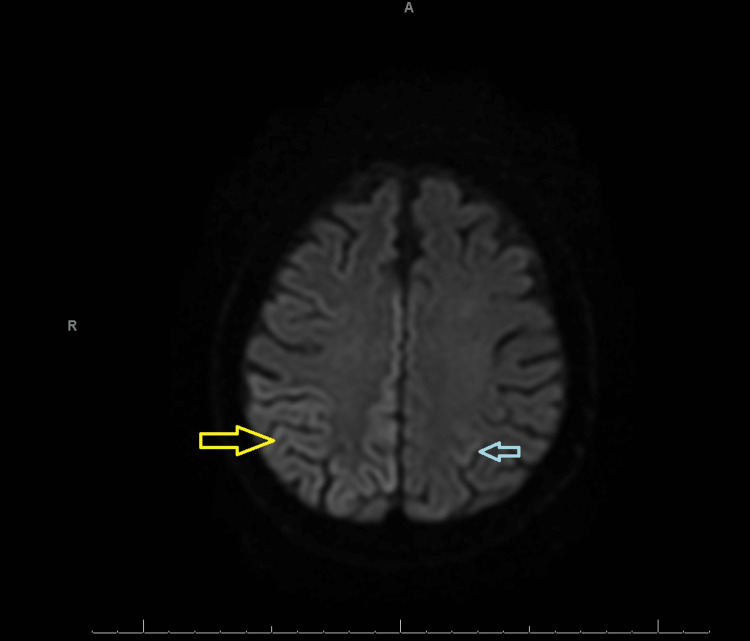
MRI of the brain without contrast (DWI) displaying areas of curvilinear hyperintense signal in the right parietal, frontal, and temporo-occipital cortex (yellow arrow*) and to a lesser extent in the left parietal cortex (blue arrow) *Yellow arrow displays the area of restricted diffusion in the right occipital cortex. DWI: Diffusion-weighted imaging.

**Figure 3:**
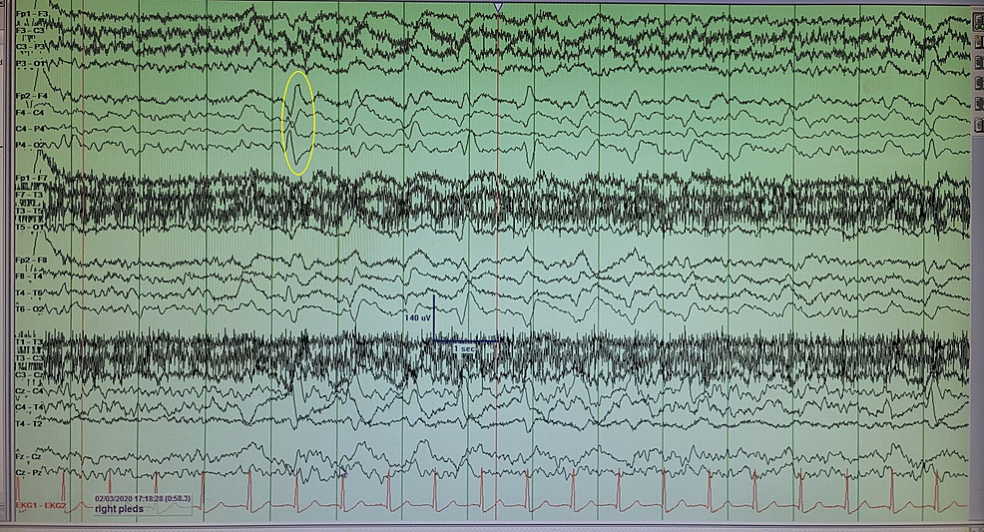
EEG displaying LPDs (yellow oval) at 0.5-1 Hz in the right hemisphere EEG: Electroencephalogram; LPD: Lateralized periodic discharges.

He underwent a second MRI without contrast, and this time, the right parietal, frontal, and temporo-occipital cortex displayed multifocal cortical restricted diffusion to a smaller extent in the left parietal cortex (Figures 1, 2). In the right hemisphere, EEG revealed continuous slowing and lateralized periodic discharges (LPDs) of 0.5-1 Hertz (Hz) as well as generalized periodic discharges of 0.5-1 Hz (Figure 3).

He was then started on antiepileptic drugs, specifically Keppra 1000 mg twice a day, to see if the seizures were the cause of his overall clinical picture. His antiseizure drugs were adjusted to Keppra 1500 mg twice a day with the addition of Depakote 2000 mg twice a day and Lacosamide 50 mg daily because the workup was ongoing and because of continuing LPDs on extended EEG. His uncontrollable seizures led to the conclusion that his condition was a symptom of a more serious neurological condition. The patient needed intubation and sedation for respiratory compromise in the interim because antiseizure medication had not resulted in any appreciable clinical improvement. His presentation heightened clinical suspicion for RPD including CJD and reversible causes such as autoimmune-mediated encephalitis, paraneoplastic, toxic-metabolic, neurodegenerative, and infectious pathologies.

The results of the cerebrospinal fluid (CSF) examination were unremarkable other than a slightly high protein level of 71.5 mg/dl (normal range: 15-60 mg/dL) and neuron-specific enolase of 20.4 ng/mL. Particular protein, including protein 14-3-3, was negative.

He was started on high-dose intravenous methylprednisolone for five days with no discernible improvement due to a strong suspicion of autoimmune encephalitis. His hospitalization was further worsened by persistent respiratory failure, bacteremia, and clinical decline till his death. The family consented to an autopsy, which confirmed the sporadic CJD diagnosis.

## Discussion

A sporadic case of CJD can have a definite, probable, or possible diagnosis. As implied by the term, definitive sporadic CJD diagnostic criteria are met if scrapie-associated fibrils are present and the disease is identified using standard neuropathological methods, immunocytochemistry, or Western blot confirmation of protease-resistant PrP [1,5]. Conversely, probable and possible diagnoses are exclusionary diagnoses. For a probable sporadic CJD, the following criteria should be met: neuropsychiatric disorder and positive real-time quaking inversion recovery (RT-QuIC) in CSF [6] or other tissues or RPD; at least two of the following four clinical features: myoclonus, visual or cerebellar signs, pyramidal/extrapyramidal signs, and akinetic mutism; and a positive result on at least one of the following laboratory tests: a typical EEG (periodic sharp wave complexes) during an illness of any duration, a positive 14-3-3 CSF assay in patients with a disease duration of fewer than two years, or a high signal in caudate/putamen on MRI brain scan or at least two cortical regions (temporal, parietal, and occipital) either on diffusion-weighted imaging (DWI) or fluid-attenuated inversion recovery (FLAIR) [7]. Possible CJD diagnostic criteria include progressive dementia and at least two of the four clinical features described, the absence of any positive test results mentioned above, and a duration of illness of fewer than two years.

Patients with RPD that are later diagnosed with CJD range from 20% to 80%, which may suggest their premature reference to higher centers without prior testing [8-10]. Nonetheless, a case of RPD should be taken seriously with a detailed history and examination. It is also noted that more than half of RPD mimickers are later diagnosed with another neurodegenerative disease [8]. A classic case of CJD can present with non-specific symptoms such as headache, malaise, and dementia versus rapidly declining cognition, behavioral changes, mutism, and startle-induced myoclonus. Seizures as the primary presentation are seldom encountered in these patients. Literature has reported a few cases of CJD with status epilepticus as an atypical presenting feature of this fatal neurodegenerative disease. There have been multiple case reports where the authors have defined seizures in CJD on a per-case basis. Some authors have defined seizures as an electrographic pattern with clinical correlate versus others who described it based on just EEG seizure patterns without clinical relation [11]. Thus, it may become challenging to decide whether to treat the electrographic seizures solely. Alternatively, physicians and intensivists should also pay attention to the fact that this type of atypical case requires judicious care to treat relentless seizures.

CJD poses a significant diagnostic challenge, even in this modern world. Physicians look for a combination of neuropsychiatric symptoms, EEG, MRI brain, and CSF markers of neuronal damage, such as 14-3-3 and tau protein, to determine CJD. Recently, a novel test namely RT-QuIC has aided in the better determination of the disease process. RT-QuIC is an amplification assay that can detect small amounts of scrapie-associated prion protein (PrP^sc^)^ ^in the CSF. PrP^sc^ binds to recombinant PrP in the CSF, which induces a change in the structure of PrP^sc^ to form fibrils. The aggregates of fibrils are then examined to evaluate their binding with the fluorescent dye thioflavin T. This fluorescence is then detected in real time [6].

In a systematic review, evaluating the diagnostic value of 14-3-3 and RT-QuIC, the authors found that both tests had similar sensitivity (88% and 86%, respectively), but RT-QuIC had a higher specificity at 99.5% compared to 80% in 14-3-3 [7]. Of note, nasal brushing RT-QuIC had the highest sensitivity and specificity and thus may be used in the future as a superior diagnostic tool, but more studies are needed regarding this specific technique [6,7]. Classic MRI findings have a high diagnostic value as well as a sensitivity of 92%-96% and a specificity of 93%-94% [8],^ ^while classic findings on EEG are found in approximately two-thirds of cases.

## Conclusions

As autoimmune encephalopathies, repeated strokes, seizures, and cerebral vasculitis are common causes of RPD; it is crucial to rule out all other probable causes and treat any reversible ones in order to accurately assess a patient with RPD. The diagnostic criteria for CJD must be approached with care by physicians because an autopsy is the only way to make a definitive diagnosis. Depending on the degree of suspicion for CJD and taking into account the disease's several unusual manifestations, ancillary tests should be undertaken. These standards ought to guarantee a more precise diagnosis of sporadic CJD.
